# A Review of Notch Processing With New Insights Into Ligand-Independent Notch Signaling in T-Cells

**DOI:** 10.3389/fimmu.2018.01230

**Published:** 2018-06-01

**Authors:** Martin Peter Steinbuck, Susan Winandy

**Affiliations:** Immunology Training Program, Department of Pathology and Laboratory Medicine, Boston University School of Medicine, Boston, MA, United States

**Keywords:** Notch, T-cell, endocytosis, ligand-independent, T-cell receptor, protein kinase C

## Abstract

The Notch receptor is an evolutionarily highly conserved transmembrane protein essential to a wide spectrum of cellular systems, and its deregulation has been linked to a vast number of developmental disorders and malignancies. Regulated Notch function is critical for the generation of T-cells, in which abnormal Notch signaling results in leukemia. Notch activation through *trans*-activation of the receptor by one of its ligands expressed on adjacent cells has been well defined. In this canonical ligand-dependent pathway, Notch receptor undergoes conformational changes upon ligand engagement, stimulated by a pulling-force on the extracellular fragment of Notch that results from endocytosis of the receptor-bound ligand into the ligand-expressing cell. These conformational changes in the receptor allow for two consecutive proteolytic cleavage events to occur, which release the intracellular region of the receptor into the cytoplasm. It can then travel to the nucleus, where it induces gene transcription. However, there is accumulating evidence that other pathways may induce Notch signaling. A ligand-independent mechanism of Notch activation has been described in which receptor processing is initiated *via* cell-internal signals. These signals result in the internalization of Notch into endosomal compartments, where chemical changes existing in this microenvironment result in the conformational modifications required for receptor processing. This review will present mechanisms underlying both canonical ligand-dependent and non-canonical ligand-independent Notch activation pathways and discuss the latter in the context of Notch signaling in T-cells.

## Introduction

The Notch receptors are evolutionarily highly conserved transmembrane proteins essential to a wide spectrum of cellular systems (1). Notch signaling is especially important for normal thymic T-cell development (2–5) and remains crucial after the release of T-cells into the periphery (6). Thus, it is not surprising that deregulation of the Notch signal can result in T-cell acute lymphoblastic leukemia (T-ALL) in mice and humans (7, 8).

The earliest lymphocyte progenitors that migrate to the thymus are provided with Notch ligands by the thymic microenvironment, initiating the T-cell program while preventing the B-cell fate (2, 3). It also has been reported that Notch signals are important in subsequent T-cell fate decisions that occur in the thymus [reviewed in Ref. (9)], including αβ vs. γδ (10–14) and CD4 vs. CD8 lineage choices (15, 16). A role for Notch has been described in peripheral T-cells as well, where it has been linked to 1) T-helper (T_H_) cell lineage development and cytokine gene expression (6, 17–24), 2) inducible regulatory T-cell development (25), 3) regulatory T-cell survival and function (26–29), 4) differentiation of CD8^+^ T-cells into terminal effector vs. memory cells (6), and 5) proliferation and survival of T-cells (24, 30–35).

The Notch signaling pathway is unique as Notch is a transcriptional regulator initially expressed as a membrane-bound cell surface receptor. Notch activity is regulated at the level of proteolytic processing of the membrane-bound form to allow release of the active intracellular fragment. In mature T-cells, Notch processing can be triggered *via* two different mechanisms. First, well-defined canonical Notch-ligand-dependent modes have been reported, whereby Notch ligand is expressed on the surface of interacting antigen-presenting cells. However, many groups have reported that Notch can also be activated by T-cell receptor (TCR) complex/CD28 signaling pathways, a much less well-defined process that can occur in the absence of ligand (24, 31–33, 36, 37). Investigation into this unique role of Notch activation is still in its infancy. Our recent report provides arguably the first evidence that ligand-independent Notch activation is required for optimal T-cell proliferation and activation (37). In this review, we will discuss what is known about how Notch processing is regulated, and how these studies, together with our recent report, provide insight into the mechanism underlying this novel activation pathway.

## Deregulation of Notch Function is Dangerous to T-Cells

**Figure 1:**
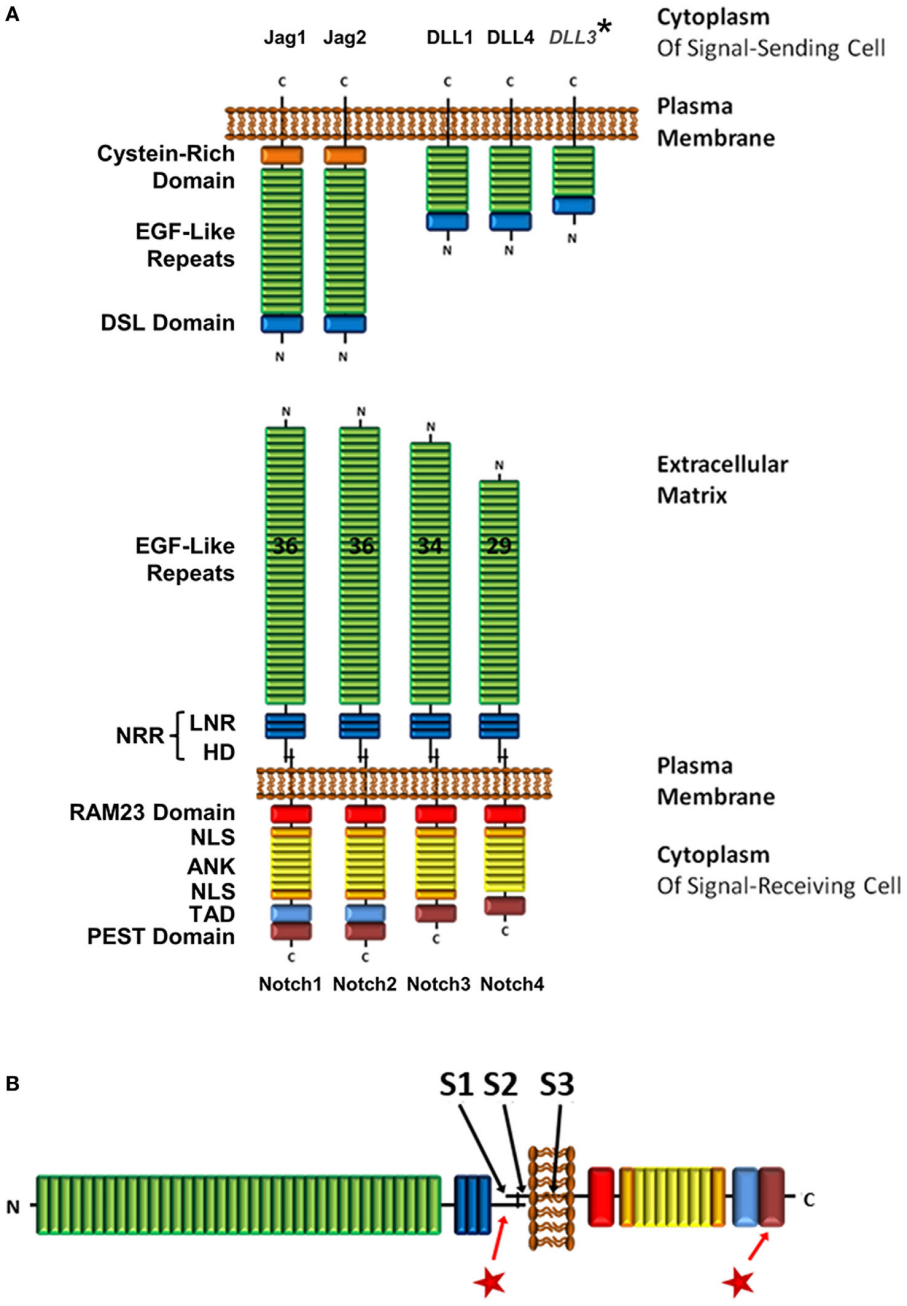
Structure of Notch receptor family and its ligands. **(A)** Structure of Notch receptors on signal-receiving cells and Notch ligands on signal-sending cells. In humans and mice, there are four Notch receptors, and five Notch ligands within two families, the Jagged and the DLL family. *However, DLL3 is expressed exclusively in intracellular compartments. **(B)** Locations of the three cleavage sites on the Notch receptor (S1–3), and mutation hotspot regions in the HD and PEST domains 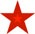. Abbreviations: NRR, negative regulatory region; LNR, cysteine-rich Lin12/Notch repeats; HD, heterodimerization domain; NLS, nuclear localization sequence; TAD, transcriptional activation domain; DSL, Delta/Serrate/Lag-2; Jag, jagged; DLL, delta-like.

Precise regulation of Notch signaling is crucial. Deregulated gain of Notch1 function has been implicated in more than 60% of T-ALL patients, making this an important mutation in leukemogenesis (38). *Notch1* mutations are generally located in two hotspots (Figure 1B), which gives insight into how Notch1 function is regulated (38). The most common mutations are located in exons 26 and 27, which code for the heterodimerization domain (HD), a region that is essential in the regulation of Notch activity as discussed in the next section. These mutations destabilize the HD domain and result in loss of autoinhibition (39). Consequently, the receptor is constitutively activated. The second hotspot is located in exon 34, which codes for the PEST [rich in proline (P), glutamic acid (E), serine (S), and threonine (T)] domain. Here, mutations generally cause truncations, most commonly by generating premature stop codons, resulting in deletion of the domain (40). The PEST domain is essential to targeting rapid degradation of the activated Notch protein, and its deletion results in an extended signaling half-life.

## Structure of Notch Receptors and its Importance to Regulation of Function

The Notch family of type-1 transmembrane receptors consists of four protein paralogs (Notch1–4) in humans and mice (Figure 1A), with mostly non-redundant functions. T-cells express Notch1, 2, and 3 (41–43). Before integration into the plasma membrane, the Notch receptor is post-translationally cleaved at the S1 site (Figure 1B), which is located 70 amino acids (aa) N-terminal of the transmembrane domain. This cleavage occurs inside the *trans*-Golgi network by a furin-like protease, resulting in a heterodimer that is held together by Ca^2+^-dependent ionic bonds (44, 45). The two polypeptides that constitute the mature membrane-bound form of Notch are called the extracellular domain (ECD) and the transmembrane fragment (TMF). While the ECD is exclusively extracellular, the TMF is comprised of a small 70aa extracellular portion, the transmembrane domain and an intracellular domain.

Starting at the N-terminus, the ECD consists of 29–36 epidermal growth factor (EGF)-like domains, of which some are calcium-binding (cbEGF). cbEGF12 (as counted from the N-terminus) is reported to be the main binding domain involved in receptor–ligand interactions; however, additional EGF sites may contribute to increase binding stability (46, 47).

Following the EGF-like domains is the negative regulatory region (NRR), which encompasses three cysteine-rich Lin12/Notch repeats (LNR) and the fragment-spanning HD domain that results from S1 cleavage, and connects the TMF and ECD polypeptides to form the Notch heterodimer (Figure 1A) (44). The NRR is crucial in preventing Notch activation in the absence of the correct signal (48–50). Upon receptor–ligand interaction, conformational changes in the NRR allow access by ADAM proteins to the S2 cleavage site (Figure 1B). This site is located 12aa away from the membrane in the extracellular region of the TMF and is usually masked by the LNR (51). In leukemia, mutations in the HD domain either elongate the sequence between the S2 site and the LNR or destabilize the region *via* point mutations, small insertions, or short deletions (Figure 1B). These sequence changes prevent the NRR from auto-inhibiting Notch activation, which ultimately leads to unregulated Notch signaling (39).

Within the TMF, C-terminal of the HD domain, is the transmembrane domain. It contains the S3 cleavage site (Figure 1B), which is a substrate for regulated intramembrane proteolysis by the γ-secretase complex (γSec) (52). This event will occur only after the rate-limiting S2 cleavage has taken place, making S3 accessible to γSec (53). S3 proteolysis results in the release of the Notch intracellular domain [hereafter referred to as intracellular Notch (ICN)] from the membrane and allows Notch signaling to be initiated.

Canonical Notch ligands of the Delta/Serrate/Lag-2 (DSL) family in humans and mice fall into one of two classes, depending on whether they are a homolog of the *Drosophila* Notch ligand Delta or Serrate (Figure 1A). The Delta-like (DLL) proteins include DLL1, DLL3, DLL4, and the Serrate homologs are comprised of Jagged1 (Jag1) and Jag2. Even though functional differences have been ascribed to the four Notch receptors, ligation with either DLL or Jagged family ligands leads to the activation of the same canonical signaling pathway (54). Although Notch can be activated in T-cells through interaction with canonical Notch ligands on adjacent cells (e.g., DLL4, and to a lesser extent DLL1 and Jag2, on dendritic cells, as well as Jag1 on B-cells) (12, 19, 55, 56), it has also been demonstrated by many groups that TCR/CD28 signaling alone is able to initiate Notch cleavage and activation (24, 31–33, 36, 37). In order to understand the mechanism underlying this activation process, a closer look at how Notch receptor is processed *via* canonical ligand-dependent pathways is warranted.

## Mechanisms Underlying Ligand-Induced Notch Processing

**Figure 2:**
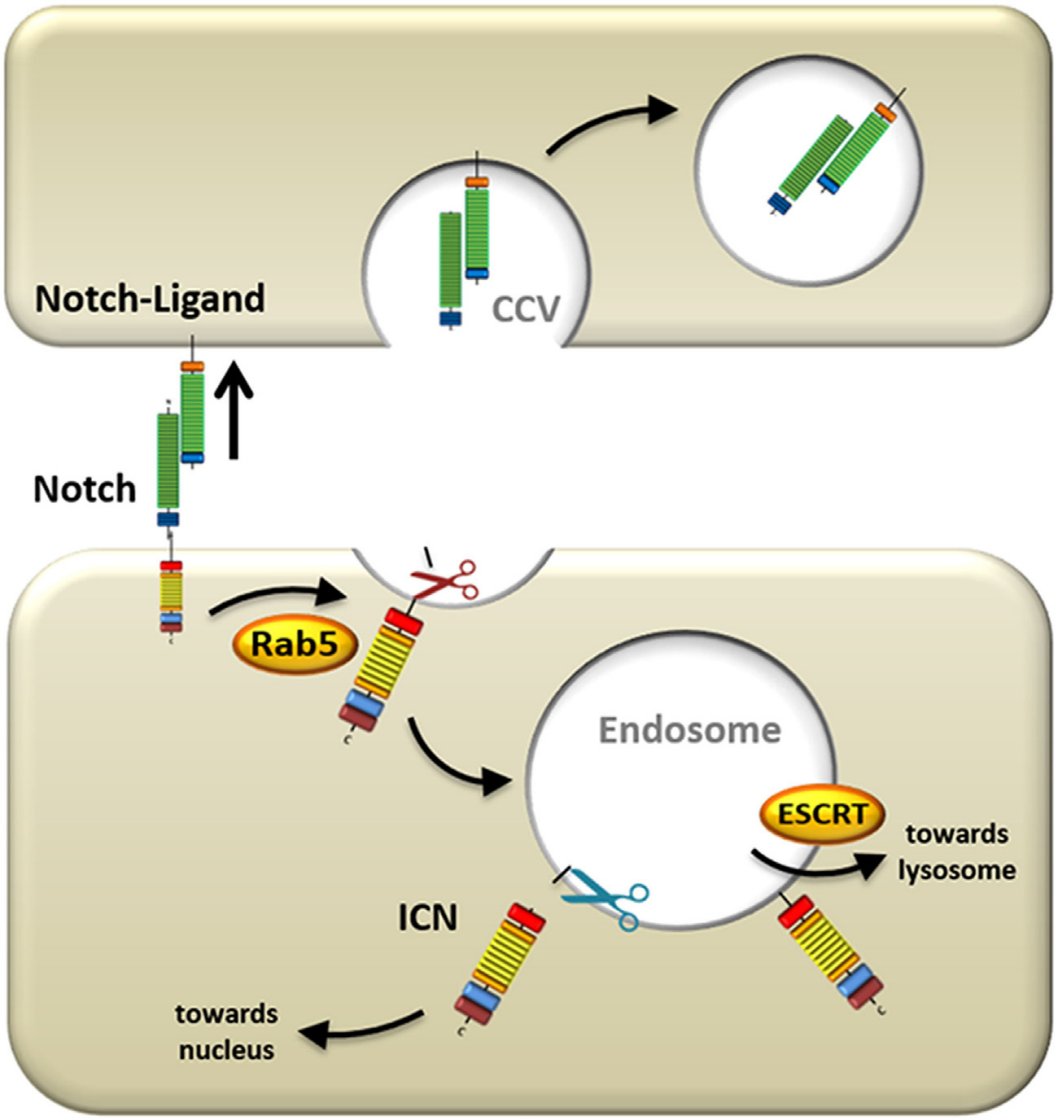
Mechanisms underlying ligand-induced Notch processing. Upon ligand engagement, the signal-sending cell exerts a pulling force on the Notch receptor by internalizing the ligand and Notch extracellular domain. Simultaneously, the residual portion of the Notch receptor is endocytosed into the signal-receiving cell *via* Ras-related protein 5 (Rab5)-positive vesicles, where it is cleaved by ADAMs (red scissors). This first cleavage event primes the Notch receptor for a subsequent cleavage by γ-secretase complex (blue scissors) in the endosome, which releases intracellular Notch (ICN) from the membrane and allows it to transmigrate to the nucleus. Alternatively, the Notch receptor can be escorted by endosomal sorting complexes required for transport proteins (ESCRT) to the lysosome where it is degraded.

The mechanism of Notch activation that has been studied most thoroughly is initiated by the canonical ligands described above. Since Notch activation is auto-inhibited through its NRR domain (49), which masks the S2 site and prevents ADAM-mediated cleavage, conformational changes in the receptor need to take place before Notch can become processed. In the secondary structure of the rod-shaped Notch receptor, the NRR and the ligand-binding EGF domains are spatially far apart from each other. Therefore, it is unlikely that allosteric regulation of the receptor could produce the necessary conformational changes in the heavily folded NRR region to initiate Notch signaling. Using *Drosophila* imaginal disk and retinal cells with defective dynamin function, important for endocytic vesicle formation, it was shown that Notch activation is dependent on ECD dissociation from the receptor and *trans*-endocytosis into the ligand-expressing cells (Figure 2) (57). It has been proposed that this pulling force causes conformational changes in the NRR region, which consequently allows S2 cleavage to occur (58), after which the ECD is free to be *trans*-endocytosed. A study in murine C2C12 cells also demonstrated the requirement of Notch ECD *trans*-endocytosis and substantiated that ligand binding alone is insufficient to activate Notch (59). However, this study also provided evidence that ECD dissociation occurs even in the presence of ADAM inhibitors. This suggests that dissociation is not a consequence of S2 site cleavage, but rather that ECD dissociation occurs first, and subsequently allows for S2 site cleavage by ADAMs (59, 60).

Once the S2 site has been exposed, further processing is facilitated by ADAMs (Figure 2). In *Drosophila*, ADAM10/Kuz has been shown to activate Notch receptors. This was demonstrated using dominant negative ADAM10 flies (61), ADAM10-deficient flies (62), and RNA interference of ADAM10 (63), all of which inhibited Notch processing and activity. Furthermore, physical contact between Notch and ADAM10 was shown by co-immunoprecipitation (63).

In addition to ADAM10, in mammalian cells, ADAM17/TACE also can process Notch (64, 65). However, ADAM10 and ADAM17 are differentially involved in Notch processing. Using multiple approaches of Notch activation in either ADAM10- or ADAM17-defective cells, it was shown that ADAM10 is absolutely required for Notch processing upon ligand engagement in mammalian cells (66). ADAM17 was not able to rescue the ADAM10-deficient phenotype, and dominant negative ADAM17 expression did not inhibit ligand-induced Notch processing. By contrast, ADAM17 was able to process Notch under conditions of ligand-independent activation including EDTA chelation, or when using Notch constructs that harbor a mutated NRR domain, which renders them hyperactive. Thus, it was concluded that ADAM17 is necessary for ligand-independent Notch processing, whereas ADAM10 is responsible for ligand-induced Notch activation. While it has yet to be determined how preferential cleavage by ADAM10 or ADAM17 occurs, it is interesting to speculate that this selective requirement might suggest that the S2 cleavage event occurs in different compartments during ligand-dependent vs. ligand-independent Notch activation.

After S2 processing by the ADAM proteins, the final step in activating Notch and releasing ICN from the membrane is S3 cleavage mediated by γSec (Figure 2). This aspartyl protease is an intramembranously cleaving enzyme complex that is comprised of multiple protein subunits including nicastrin, anterior pharynx defective-1, presenilin enhancer-2, and the catalytically active subunit presenilin. γSec substrates generally need to be primed by ectodomain shedding from larger precursor proteins (53), such as is the case in S2-cleavage of Notch, or α-/β-secretase-mediated cleavage of amyloid precursor protein (APP) (67). Shedding of the Notch ECD results in a residual ectodomain of 12aa in length (51) and allows γSec substrate recognition *via* the nicastrin subunit that docks to the new N-terminus of Notch (68).

Although it has been suggested that γSec is present at the plasma membrane as a fully functional and active complex that can cleave APP and Notch (69, 70), γSec also localizes to early and late endosomes (LE) (71), where it processes p75 neurotrophin receptor (72), as well as APP (73). In addition, γSec can be found in lysosomes, where it experiences enhanced activity because of the low pH in the endolysosomal compartment (74). It has even been proposed that in *Drosophila* (75) as well as in mammalian cells (76) Notch/γSec co-localization to the endocytic compartment is absolutely critical to Notch activation. Overall, these data indicate that complete processing of Notch may require internalization of the receptor where it then becomes cleaved and fully activated in the endosome.

In addition to the ligand-induced mechanisms of Notch activation described above, a much less well-understood mechanism of activation has been observed in T-cells, in which TCR/CD28-stimulation is capable of activating Notch (24, 31–33, 36, 37). Notch receptors have been shown through immunofluorescence imaging to associate with the immunological synapse (IS) (33, 77) and, in the absence of Notch ligands, may be activated as bystanders of TCR stimulation. Within the crowded IS, it has been hypothesized that activation could be mediated through undefined mechanical forces acting upon Notch, causing a destabilization of the extracellular region of the receptor. Alternatively, signals downstream of TCR/CD28 may activate the IS-recruited Notch receptor. T-cell stimulation with phorbol 12-myristate 13-acetate (PMA) and ionomycin leads to robust Notch activation, suggesting that the latter is the case (37).

## The Role of Receptor Endocytosis in Notch Activation

Initially, it was believed that the sole function of endocytosis consisted of terminating plasma membrane signals by either sequestering membrane receptors from ligand binding, or by internalization and degradation of active receptor/ligand complexes [reviewed in Ref. (78)]. But it is becoming increasingly apparent that endocytosis and signaling are intertwined processes that can affect each other reciprocally [reviewed in Ref. (79, 80)]. There are multiple mechanisms of endocytosis, but possibly the most common and best studied form is clathrin-dependent [reviewed in Ref. (81)]. Clathrin is recruited to the plasma membrane by a large variety of adaptor and accessory proteins that in turn adhere to lipid- or protein-binding domains at the membrane (82). These adaptors cause clathrin polymerization into curved lattices called clathrin-coated pits. These pits continue to invaginate with the help of bending-proteins, such as epsin (83), and form clathrin-coated vesicles (CCV) that eventually bud from the internal leaflet of the membrane (Figure 2). The budding process is facilitated by dynamin, which is a GTPase that forms a helical polymer around the neck of the CCV (84). Upon dynamin-mediated scission, the fully formed vesicle is released into the cytoplasm where it is uncoated and can then fuse with other membranes (85).

The requirement for endocytosis of Notch ligands during (and even prior to) receptor engagement is well characterized [Figure 2; (86)]. However, the need for internalization of Notch itself on the signal-receiving cell as a prerequisite for signal transduction is less well established. Evidence in HeLa cells suggests that Notch endocytosis is not necessary for its activation, but instead promotes attenuation of Notch signal by reducing its expression on the cell surface (87). Notch can indeed be marked for degradation *via* ubiquitination by E3-ligases such as AIP4/Itch (88) or Nedd4 (89). It is then endocytosed with the help of Numb that recruits the AP2-clathrin adaptor-complex (90). But ubiquitination and endocytosis are by no means exclusively linked to the downregulation of Notch signaling.

On the contrary, the majority of data in *Drosophila* support a crucial role for endocytosis in activation of Notch. The first findings implicating endocytosis in Notch activation were studies using dynamin-defective *shibire-*mutants, in which deletion of dynamin in signal-receiving cells disrupted Notch signal induction (91). More recently, detailed studies of Notch localization and activity were conducted, in which successive steps of the endocytic pathway were selectively blocked in *Drosophila* imaginal disks and oocytes (75). Deletion of *shibire* (a dynamin ortholog), Ras-related protein 5 (Rab5; Figure 2) and Avalanche (the latter two are proteins that regulate entry of cargo into the early endosome), resulted in Notch accumulation at or just below the plasma membrane and significantly reduced Notch signaling as measured by a *LacZ* reporter assay. However, blocking the endocytic pathway at later stages by deleting “endosomal sorting complexes required for transport proteins (ESCRT),” which control sorting into LE (Figure 2), or Fab1 that is important in (pre)-lysosomal compartments, did not attenuate Notch signaling, but instead elevated it (75). This enhanced signaling may be a result of prolonged retention of Notch in an environment where it can be processed by γSec. In mammalian cells, it was demonstrated that Notch1 is endocytosed during ligand-dependent activation (60). This internalization is dependent on clathrin and dynamin resulting in the presence of Notch receptor in early Rab5-negative endosomal vesicles.

Further evidence placing Notch processing in the endosome comes from experiments in *Drosophila* using defective variants of the vacuolar proton pump V-ATPase (92, 93) and in mammalian cells by pharmacological inhibition of vacuolar H^+^ ATPase (94), which prevent the acidification of the endosome. In both cases, Notch activation was attenuated. This, together with data showing that γSec operates optimally in an acidic environment (74), suggests that Notch is preferentially processed in the endosome by γSec (Figure 2), and that internalization does play an important role in Notch activation.

**Figure 3:**
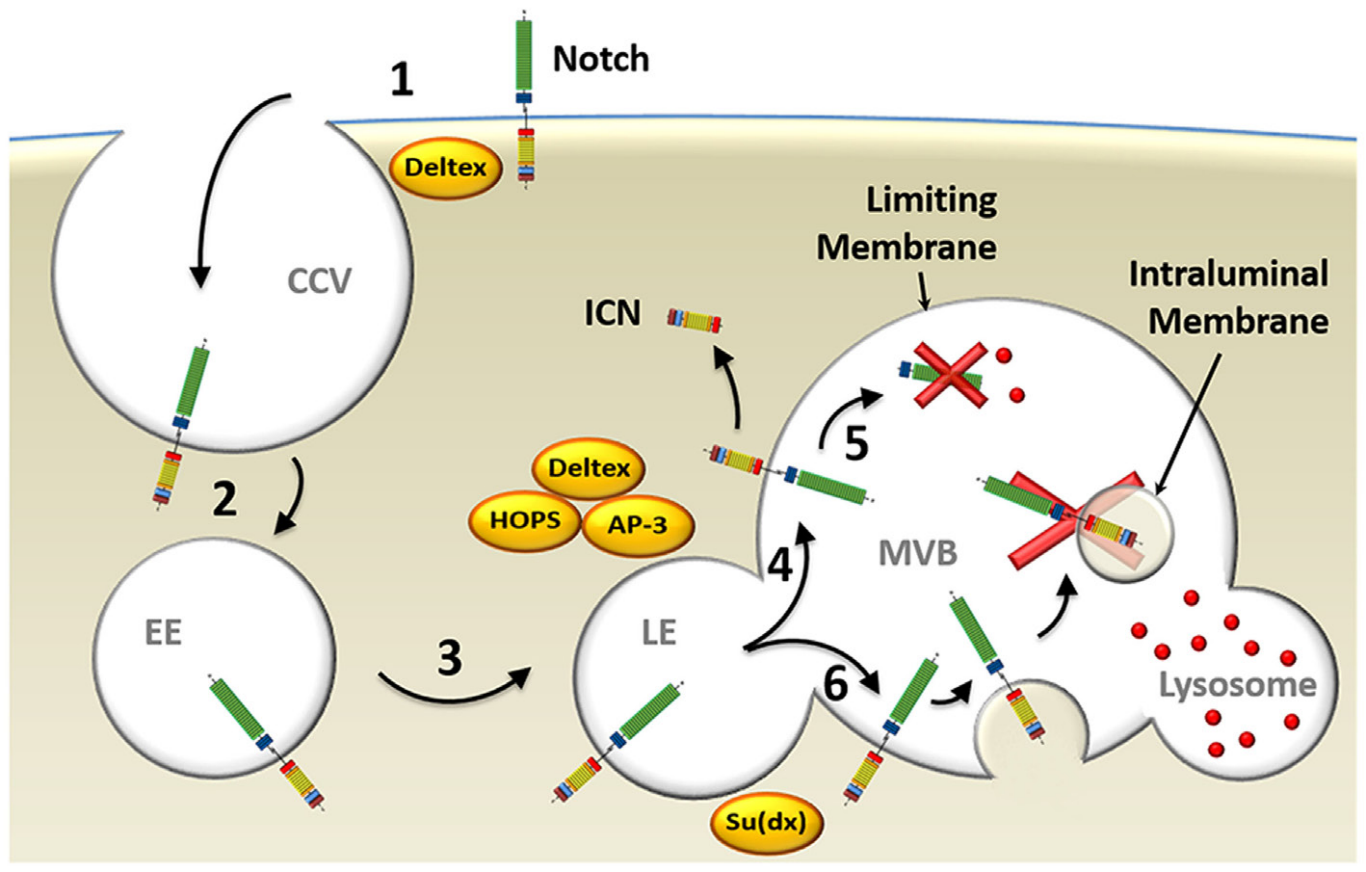
Model of endocytosis in ligand-independent Notch activation. (1) Deltex facilitates ligand-independent Notch receptor internalization into clathrin-coated vesicles (CCV) (2) that fuse with Ras-related protein 5-positive early endosomes (EE). (3) Deltex forms a complex with adaptor protein 3 (AP-3) and homotypic fusion and vacuole protein sorting (HOPS) that directs Notch to Rab7-positive late endosomes (LE) and (4) targets it to the limiting membrane of the multivesicular body (MVB). (5) This protects intracellular Notch (ICN) from lysosomal degradation and allows its release into the cytosol upon γ-secretase complex-mediated processing. (6) Alternatively, Su(dx) can redirect Notch into the intraluminal vesicles, where the Notch receptor will be proteolyzed when the MVB fuses with a lysosome.

In ligand-independent receptor activation, the E3 ubiquitin ligase Deltex is implicated in the regulation of Notch endocytic trafficking in *Drosophila* (Figure 3). This was demonstrated with null mutations for Deltex that led to failure of Notch internalization and activation (95), as well as overexpression experiments that strongly enhanced Notch signaling (96). Deltex stabilizes the receptor in the endocytic compartment allowing signal transduction to proceed, as assayed by expression of Notch-reporter genes (97, 98). Deltex also forms a complex with adaptor protein 3 [AP-3; (99)] and “homotypic fusion and vacuole protein sorting” [HOPS; (100)], both of which deliver Notch to the exterior membrane of the multivesicular body (MVB) called the limiting membrane. This localization on the exterior membrane places the intracellular domain of Notch in the cytosol, where, upon its cleavage to form ICN, it is free to translocate to the nucleus and promote activation of its target genes (101). Supporting this model are studies carried out in *Drosophila* examining the role of “Suppressor of Deltex” [Su(dx)], also an E3 ubiquitin ligase, which directly opposes the function of Deltex. Su(dx) facilitates Notch incorporation into the membranes of intraluminal vesicles located inside the MVB, instead of localization to the limiting membrane, and prevents ICN signaling. This results in ICN being spatially sequestered from the cytosol and degraded (101). Interestingly, HOPS and AP-3 are not needed in ligand-dependent processing of Notch indicating that separate pathways underlie ligand-dependent and ligand-independent Notch activation (101).

Overall, it can be concluded that, at least in some forms of Notch activation, endocytosis of the receptor and its shuttling through the endosomal compartment are important factors. In the ligand-independent process, Deltex facilitates the endocytosis of Notch from the plasma membrane and, together with HOPS and AP3, protects it from degradation by targeting it to limiting membranes of the MVB. There, it can be processed by γSec allowing release of ICN into the cytosol.

Whereas, Deltex proteins are not required for thymic T-cell development (102), in which Notch activation is largely dependent on DLL4 (103), we hypothesize that in systems that utilize ligand-independent Notch activation, such as that initiated by TCR/CD28-mediated stimulation, Deltex, as well as many of the other proteins in the endocytic pathway discussed above, will be important players.

## Endogenous Processing of Endocytosed Receptor

**Figure 4:**
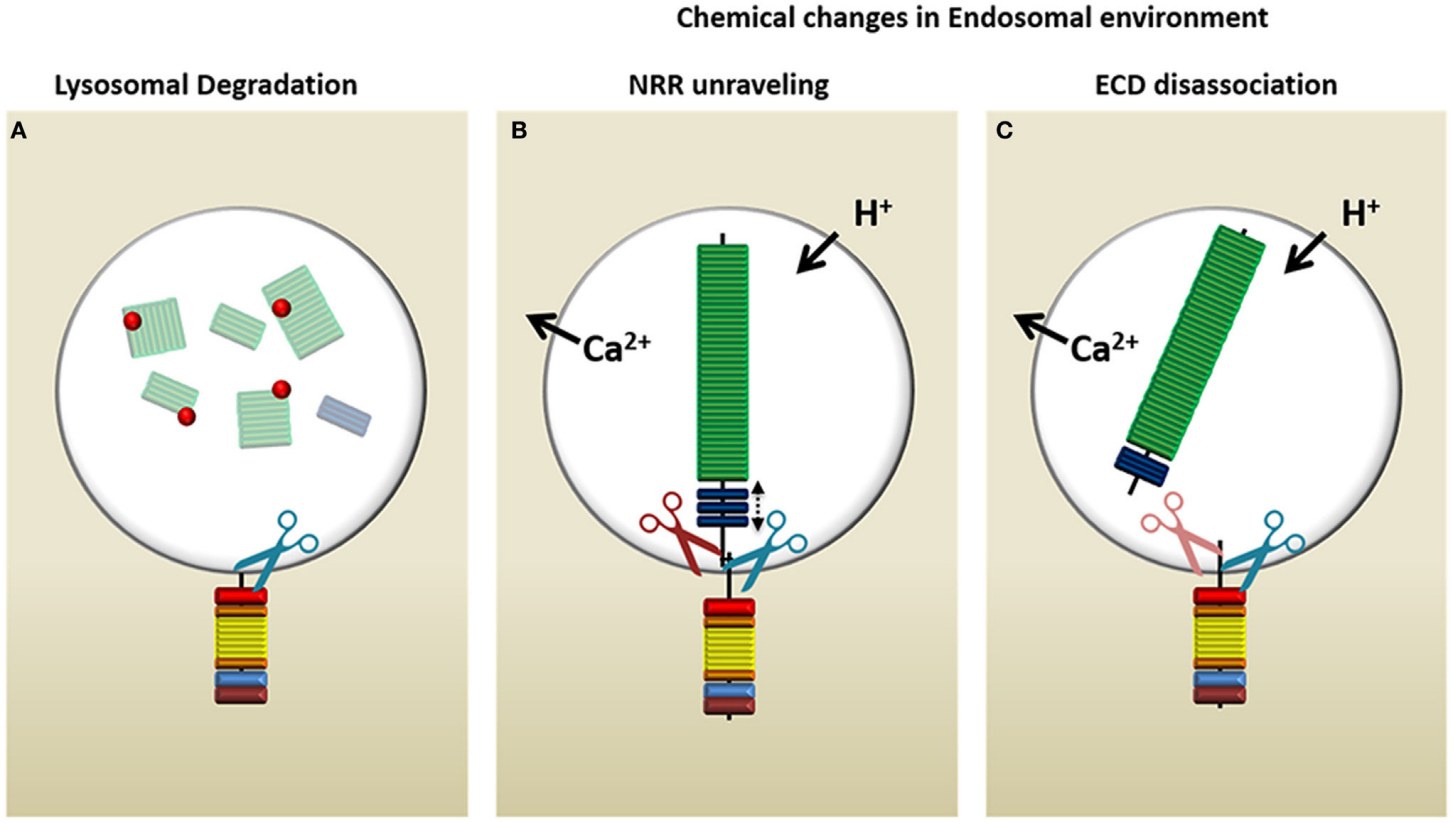
Possible mechanisms of Notch processing in the endosome. Upon delivery of Notch to the multivesicular body, there are three possible ways in which the endocytic environment may prepare the Notch receptor to allow the release of intracellular Notch from the limiting membrane. **(A)** Lysozymes (red dots) present in this compartment may proteolyze the intraluminal region of Notch and therefore allow γ-secretase complex (γSec) cleavage to proceed. **(B)** The change in ion concentrations and decreasing pH may cause the negative regulatory region (NRR) of Notch to unravel, mimicking a ligand-mediated pull, which opens the S2 site for access by ADAM (red scissors), followed by γSec cleavage (blue scissors). **(C)** The lysosomal environment may cause the Notch extracellular domain (ECD) to dissociate entirely, in which case γSec may directly process the 70aa juxtamembrane stub or rely on ADAM proteases (light red because of uncertain requirement) to increase its affinity for the S3 site through processing the juxtamembrane stub to 12aa.

Canonical ligand-mediated Notch activation relies on conformational changes in the receptor that are induced by the ligand to unmask the protected S2/S3 sites for cleavage by ADAMs and γSec. In ligand-independent Notch activation, these changes must be driven by another method. There are three possible scenarios by which this process might occur within an endosomal microenvironment [Figure 4; (104)]. First, lysozymes may proteolyze the Notch-ECD that extends into the intraluminal space of the MVB, thus removing inhibitory sequences that prevent γSec recognition (Figure 4A). Whereas this seems to be the simplest answer, it would circumvent any rate-limiting checkpoints of Notch processing (i.e., ADAM-mediated cleavage of S2) and expose Notch to constitutive activation by γSec (105). This would result in Notch signaling that is difficult to regulate. The other two possibilities involve naturally occurring changes in the LE microenvironment. Specifically, adjustments in ion concentrations (106, 107), especially those of Ca^2+^ that are important in NRR and HD stability, and/or the gradual acidification of the endosome could result in (1) destabilization of the NRR and unmasking of the S2 site (Figure 4B) or (2) full dissociation of the ECD (Figure 4C). Whereas NRR destabilization would mimic a ligand-induced pull, which then still requires ECD removal by ADAM before γSec can recognize Notch, the immediate dissociation of the ECD at the HD domain would leave only a 70aa residual sequence. γSec has been shown to recognize substrates providing the juxtamembrane sequence is <200aa in length (53). However, the longer the sequence, the lower the affinity of γSec to its substrate, suggesting that further processing by ADAM still may be required. Even though ADAM10 and ADAM17 are predominantly expressed at the cell surface—constitutively and upon activation, respectively (108)—intracellular activity of ADAM has been documented (109, 110). Conversely, the acidic optimum for γSec activity may enable it to simply bypass S2 cleavage and directly process the 70aa-stub moiety of Notch (74).

## A Model of TCR-Activated Notch Signaling

**Figure 5:**
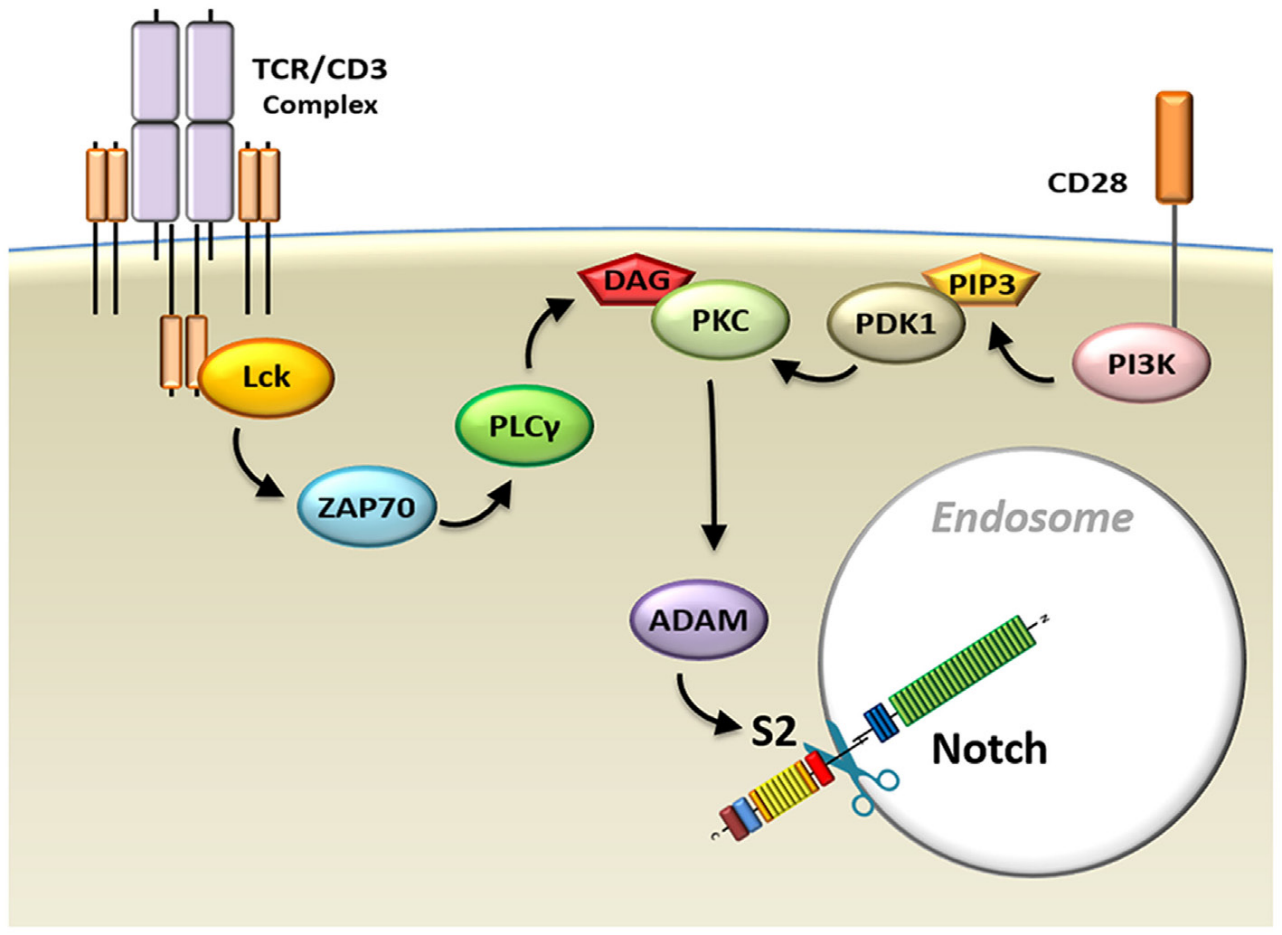
Model schematic diagram of TCR/CD28-induced Notch activation in T-cells. Signals from the TCR–CD3 complex, as well as CD28 co-receptors, activate the Notch cleavage machinery and induce endocytosis of the Notch receptor. Abbreviations: Lck, lymphocyte-specific protein tyrosine kinase; ZAP70, z-chain associated protein kinase 70 kDa; TCR, T-cell receptor.

Ligand-independent Notch processing has been described in *Drosophila* cells. However, until recently, whether this method of Notch activation occurs during normal physiological processes in mammalian cells remained murky. We have recently reported that the processing of Notch triggered by TCR/CD28 signaling in T-cells occurs *via* a ligand-independent mechanism (24, 31–33, 36, 37). TCR and CD28 signals cooperate to initiate two processes required for ligand-independent Notch activation (37). The first process consists of internalization of the receptor. Chemical adjustments in the endocytic compartment substitute for the mechanical forces that are generated by conventional Notch receptor ligation, driving conformational changes in the autoinhibitory region of Notch that are required for cleavage. Concurrently, the second process activates the machinery that performs the Notch cleavage events (Figure 5).

While it is unknown what pathways trigger internalization of the Notch receptor, Notch processing post-internalization requires signals delivered through both TCR and CD28. These signals cooperate to activate protein kinase C (PKC), which is required for Notch activation. TCR complex cross-linking initiates activation of phospholipase C-gamma, which is required for generation of the membrane anchor diacylglycerol (DAG). DAG, in turn, is a crucial factor in activation of mature PKC. However, in order to be available for activation by DAG, PKC must undergo maturation events that occur downstream of CD28 signal. Specifically, CD28 signals trigger phosphatidylinositide 3-kinase activation, which, through the phosphorylation of PIP_2_ (phosphatidylinositol 4,5-bisphosphate), generates another membrane anchor molecule, PIP_3_ [phosphatidylinositol (3,4,5)-triphosphate]. The enzyme 3-phosphoinositide-dependent protein kinase-1 (PDK1) is recruited to the IS by PIP_3_, where it can now efficiently phosphorylate PKC, priming it for activation by TCR signals (111).

Once PKC has been activated, it induces activity of ADAM10 and ADAM17. It has recently been shown, in agreement with our study, that PKCθ regulates Notch processing downstream of TCR signal and upstream of ADAM activity (112). Cleavage of the Notch receptor at the S2 site by ADAMs is required for Notch activation. However, this proteolytic event cannot occur when the S2 cleavage site of Notch is protected by its NRR autoinhibitory domain. This is why, in the absence of ligand, the internalization of the Notch receptor becomes important. We hypothesize that the gradual acidification of the endocytic environment and efflux of calcium from the endosome (106, 107) result in conformational changes in the Notch receptor. These changes would unravel the NRR and allow activated ADAMs to initiate Notch processing, ultimately resulting in the release of ICN, which can now translocate to the nucleus to activate expression of its target genes.

## Conclusion

What is the benefit of the TCR/CD28-mediated, ligand-independent mode of Notch activation to T-cells? It may have its origin in the fact that T-cells are part of a fluid system of migrating cells. Generally, Notch receptor initiates cell fate decisions in solid tissues, in which lateral interactions with ligand-expressing cells can be sustained indefinitely. By contrast, T-cells spend some of their life cycle in circulation or percolating through secondary lymphoid organs on the search for cognate antigen (113). Since the Notch pathway does not contain an inherent amplification cascade that would enhance external stimuli—Notch signal input creates a stoichiometric signal output—the one-to-one interaction of ligand on antigen-presenting cells with Notch receptor on T-cells, as well as the short half-life of ICN, may not result in sufficient Notch signal to robustly activate a full program of Notch target gene activation. TCR/CD28 signaling, which does undergo multiple rounds of signal amplification, may serve to circumvent this deficiency and activate adequate levels of Notch signal when a cognate antigen has been recognized. Since Notch signaling optimizes T-cell responses, such as proliferation and activation, the T-cell is not reliant on additional Notch ligands and can autonomously couple Notch activation to TCR stimulation.

Whether these pathways contribute to ligand-independent Notch activation in leukemia cells is unknown. Our recent report has provided evidence that receptor endocytosis occurs even in Notch-dependent T-leukemic cell lines as a prerequisite step for Notch activation (37). Further studies will be vital to investigate the importance of this pathway to the constitutive Notch activation that drives survival and proliferation of leukemic T-cell as it could be the entryway into development of new therapies.

## Author Contributions

MS and SW wrote the paper. MS prepared the figures.

## Conflict of Interest Statement

The authors declare that the research was conducted in the absence of any commercial or financial relationships that could be construed as a potential conflict of interest.

## References

[1] Andersson ER Sandberg R Lendahl U. Notch signaling: simplicity in design, versatility in function. Development (2011) 138:3593–612. 10.1242/dev.063610 21828089

[2] Pui JC Allman D Xu L DeRocco S Karnell FG Bakkour S Notch1 expression in early lymphopoiesis influences B versus T lineage determination. Immunity (1999) 11:299–308. 10.1016/S1074-7613(00)80105-3 10514008

[3] Radtke F Wilson A Stark G Bauer M van Meerwijk J MacDonald HR Deficient T cell fate specification in mice with an induced inactivation of Notch1. Immunity (1999) 10:547–58. 10.1016/S1074-7613(00)80054-0 10367900

[4] Radtke F Wilson A Ernst B Macdonald H. The role of Notch signaling during hematopoietic lineage commitment. Immunol Rev (2002) 187:65–74. 10.1034/j.1600-065X.2002.18706.x 12366683

[5] Allman D Punt JA Izon DJ Aster JC Pear WS. An invitation to T and more: Notch signaling in lymphopoiesis. Cell (2002) 109:1–11. 10.1016/S0092-8674(02)00689-X 11983148

[6] Amsen D Helbig C Backer RA. Notch in T cell differentiation: all things considered. Trends Immunol (2015) 36:802–14. 10.1016/j.it.2015.10.007 26617322

[7] Ellisen LW Bird J West DC Soreng AL Reynolds TC Smith SD TAN-1, the human homolog of the *Drosophila* Notch gene, is broken by chromosomal translocations in T lymphoblastic neoplasms. Cell (1991) 66:649–61. 10.1016/0092-8674(91)90111-B 1831692

[8] Pear WS Aster JC Scott ML Hasserjian RP Soffer B Sklar J Exclusive development of T cell neoplasms in mice transplanted with bone marrow expressing activated Notch alleles. J Exp Med (1996) 183:2283–91. 10.1084/jem.183.5.2283 8642337 PMC2192581

[9] Deftos M Bevan M. Notch signaling in T cell development. Curr Opin Immunol (2000) 12:166–72. 10.1016/S0952-7915(99)00067-9 10712939

[10] Washburn T Schweighoffer E Gridley T Chang D Fowlkes BJ Cado D Notch activity influences the αβ versus γδ T cell lineage decision. Cell (1997) 88:833–43. 10.1016/S0092-8674(00)81929-7 9118226

[11] Doerfler P Shearman MS Perlmutter RM Presenilin-dependent γ-secretase activity modulates thymocyte development. Proc Natl Acad Sci U S A (2001) 98:9312–7. 10.1073/pnas.161102498 11470902 PMC55417

[12] Tanigaki K Tsuji M Yamamoto N Han H Tsukada J Inoue H Regulation of αβ/γδ T cell lineage commitment and peripheral T cell responses by Notch/RBP-J signaling. Immunity (2004) 20:611–22. 10.1016/S1074-7613(04)00109-8 15142529

[13] Ciofani M Knowles GC Wiest DL von Boehmer H Zúñiga-Pflücker JC Stage-specific and differential Notch dependency at the αβ and γδ T lineage bifurcation. Immunity (2006) 25:105–16. 10.1016/j.immuni.2006.05.010 16814577

[14] Maillard I Tu L Sambandam A Yashiro-Ohtani Y Millholland J Keeshan K The requirement for Notch signaling at the β-selection checkpoint in vivo is absolute and independent of the pre–T cell receptor. J Exp Med (2006) 203:2239 LP–2245. 10.1084/jem.20061020 16966428 PMC2118105

[15] Robey E Chang D Itano A Cado D Alexander H Lans D An activated form of Notch influences the choice between CD4 and CD8 T cell lineages. Cell (1996) 87:483–92. 10.1016/S0092-8674(00)81368-9 8898201

[16] Laky K Fowlkes BJ. Notch signaling in CD4 and CD8 T cell development. Curr Opin Immunol (2008) 20:197–202. 10.1016/j.coi.2008.03.004 18434124 PMC2475578

[17] Maekawa Y Tsukumo SI Chiba S Hirai H Hayashi Y Okada H Delta1-Notch3 interactions bias the functional differentiation of activated CD4+ T cells. Immunity (2003) 19:549–59. 10.1016/S1074-7613(03)00270-X 14563319

[18] Minter LM Turley DM Das P Shin HM Joshi I Lawlor RG Inhibitors of gamma-secretase block in vivo and in vitro T helper type 1 polarization by preventing Notch upregulation of Tbx21. Nat Immunol (2005) 6:680–8. 10.1038/ni1209 15991363

[19] Amsen D Blander JM Lee GR Tanigaki K Honjo T Flavell RA. Instruction of distinct CD4 T helper cell fates by different Notch ligands on antigen-presenting cells. Cell (2004) 117:515–26. 10.1016/S0092-8674(04)00451-9 15137944

[20] Tanaka S Tsukada J Suzuki W Hayashi K Tanigaki K Tsuji M The Interleukin-4 enhancer CNS-2 is regulated by Notch signals and controls initial expression in NKT cells and memory-type CD4 T cells. Immunity (2006) 24:689–701. 10.1016/j.immuni.2006.04.009 16782026

[21] Fang TC Yashiro-Ohtani Y Del Bianco C Knoblock DM Blacklow SC Pear WS. Notch directly regulates Gata3 expression during T helper 2 cell differentiation. Immunity (2007) 27:100–10. 10.1016/j.immuni.2007.04.018 17658278 PMC2801546

[22] Ong CT Sedy JR Murphy KM Kopan R. Notch and presenilin regulate cellular expansion and cytokine secretion but cannot instruct Th1/Th2 fate acquisition. PLoS One (2008) 3:e2823. 10.1371/journal.pone.0002823 18665263 PMC2474705

[23] Bailis W Yashiro-Ohtani Y Fang TC Hatton RD Weaver CT Artis D Notch simultaneously orchestrates multiple helper T cell programs independently of cytokine signals. Immunity (2013) 39:148–59. 10.1016/j.immuni.2013.07.006 23890069 PMC3762693

[24] Palaga T Miele L Golde TE Osborne BA. TCR-mediated Notch signaling regulates proliferation and IFN-gamma production in peripheral T cells. J Immunol (2003) 171:3019–24. 10.4049/jimmunol.171.6.3019 12960327

[25] Samon JB Champhekar A Minter LM Telfer JC Miele L Fauq A Notch1 and TGFbeta1 cooperatively regulate Foxp3 expression and the maintenance of peripheral regulatory T cells. Blood (2008) 112:1813–21. 10.1182/blood-2008-03-144980 18550850 PMC2518888

[26] Asano N Watanabe T Kitani A Fuss IJ Strober W. Notch1 signaling and regulatory T cell function. J Immunol (2008) 180:2796–804. 10.4049/jimmunol.180.5.2796 18292500

[27] Perumalsamy LR Marcel N Kulkarni S Radtke F Sarin A. Distinct spatial and molecular features of Notch pathway assembly in regulatory T cells. Sci Signal (2012) 5:ra53. 10.1126/scisignal.2002859 22827997

[28] Charbonnier L-M Wang S Georgiev P Sefik E Chatila TA. Control of peripheral tolerance by regulatory T cell-intrinsic Notch signaling. Nat Immunol (2015) 16:1162–73. 10.1038/ni.3288 26437242 PMC4618075

[29] Grazioli P Felli MP Screpanti I Campese AF. The mazy case of Notch and immunoregulatory cells. J Leukoc Biol (2017) 102:361–8. 10.1189/jlb.1VMR1216-505R 28292944

[30] Jehn BM Bielke W Pear WS Osborne BA. Cutting edge: protective effects of Notch-1 on TCR-induced apoptosis. J Immunol (1999) 162:635 LP–638. 9916679

[31] Dongre A Surampudi L Lawlor RG Fauq AH Miele L Golde TE Non-canonical Notch signaling drives activation and differentiation of peripheral CD4+ T cells. Front Immunol (2014) 5:54 10.3389/fimmu.2014.00054 24611064 PMC3921607

[32] Adler SH Chiffoleau E Xu L Dalton NM Burg JM Wells AD Notch signaling augments T cell responsiveness by enhancing CD25 expression. J Immunol (2003) 171:2896–903. 10.4049/jimmunol.171.6.2896 12960312

[33] Guy CS Vignali KM Temirov J Bettini ML Overacre AE Smeltzer M Distinct TCR signaling pathways drive proliferation and cytokine production in T cells. Nat Immunol (2013) 14:262–70. 10.1038/ni.2538 23377202 PMC3577985

[34] Eagar T Tang Q Wolfe M He Y Pear W Bluestone J. Notch 1 signaling regulates peripheral T cell activation. Immunity (2004) 20:407–15. 10.1016/S1074-7613(04)00081-0 15084270

[35] Rutz S Mordmüller B Sakano S Scheffold A. Notch ligands delta-like1, delta-like4 and jagged1 differentially regulate activation of peripheral T helper cells. Eur J Immunol (2005) 35:2443–51. 10.1002/eji.200526294 16047342

[36] Bheeshmachar G Purushotaman D Sade H Gunasekharan V Rangarajan A Sarin A. Evidence for a role for Notch signaling in the cytokine-dependent survival of activated T cells. J Immunol (2006) 177:5041 LP–5050. 10.4049/jimmunol.177.8.5041 17015687

[37] Steinbuck MP Arakcheeva K Winandy S. Novel TCR-mediated mechanisms of Notch activation and signaling. J Immunol (2018) 200:997–1007. 10.4049/jimmunol.1700070 29288204 PMC5854196

[38] Weng AP Ferrando AA Lee W Morris JP Silverman LB Sanchez-Irizarry C Activating mutations of NOTCH1 in human T cell acute lymphoblastic leukemia. Science (2004) 306:269–71. 10.1126/science.1102160 15472075

[39] Malecki MJ Sanchez-Irizarry C Mitchell JL Histen G Xu ML Aster JC Leukemia-associated mutations within the NOTCH1 heterodimerization domain fall into at least two distinct mechanistic classes. Mol Cell Biol (2006) 26:4642–51. 10.1128/MCB.01655-05 16738328 PMC1489116

[40] Chiang MY Xu ML Histen G Shestova O Roy M Nam Y Identification of a conserved negative regulatory sequence that influences the leukemogenic activity of NOTCH1. Mol Cell Biol (2006) 26:6261–71. 10.1128/MCB.02478-05 16880534 PMC1592797

[41] Felli MP Maroder M Mitsiadis TA Campese AF Bellavia D Vacca A Expression pattern of Notch1, 2 and 3 and Jagged1 and 2 in lymphoid and stromal thymus components: distinct ligand-receptor interactions in intrathymic T cell development. Int Immunol (1999) 11:1017–25. 10.1093/intimm/11.7.1017 10383933

[42] Fiorini E Merck E Wilson A Ferrero I Jiang W Koch U Dynamic regulation of Notch 1 and Notch 2 surface expression during T cell development and activation revealed by novel monoclonal antibodies. J Immunol (2009) 183:7212–22. 10.4049/jimmunol.0902432 19915064

[43] Koyanagi A Sekine C Yagita H. Expression of Notch receptors and ligands on immature and mature T cells. Biochem Biophys Res Commun (2012) 418:799–805. 10.1016/j.bbrc.2012.01.106 22310713

[44] Logeat F Bessia C Brou C LeBail O Jarriault S Seidah NG The Notch1 receptor is cleaved constitutively by a furin-like convertase. Proc Natl Acad Sci U S A (1998) 95:8108–12. 10.1073/pnas.95.14.8108 9653148 PMC20937

[45] Rand MD Grimm LM Artavanis-Tsakonas S Patriub V Blacklow SC Sklar J Calcium depletion dissociates and activates heterodimeric Notch receptors. Mol Cell Biol (2000) 20:1825–35. 10.1128/MCB.20.5.1825-1835.2000 10669757 PMC85363

[46] Rebay I Fehon RG Artavanis-Tsakonas S. Specific truncations of *Drosophila* Notch define dominant activated and dominant negative forms of the receptor. Cell (1993) 74:319–29. 10.1016/0092-8674(93)90423-N 8343959

[47] Hambleton S Valeyev NV Muranyi A Knott V Werner JM McMichael AJ Structural and functional properties of the human Notch-1 ligand binding region. Structure (2004) 12:2173–83. 10.1016/j.str.2004.09.012 15576031

[48] Gordon WR Vardar-Ulu D Histen G Sanchez-Irizarry C Aster JC Blacklow SC. Structural basis for autoinhibition of Notch. Nat Struct Mol Biol (2007) 14:295–300. 10.1038/nsmb1227 17401372

[49] Gordon WR Roy M Vardar-Ulu D Garfinkel M Mansour MR Aster JC Structure of the Notch1-negative regulatory region: implications for normal activation and pathogenic signaling in T-ALL. Blood (2009) 113:4381–90. 10.1182/blood-2008-08-174748 19075186 PMC2676092

[50] Gordon WR Zimmerman B He L Miles LJ Huang J Tiyanont K Mechanical allostery: evidence for a force requirement in the proteolytic activation of Notch. Dev Cell (2015) 33:729–36. 10.1016/j.devcel.2015.05.004 26051539 PMC4481192

[51] Sanchez-Irizarry C Carpenter AC Weng AP Pear WS Aster JC Blacklow SC. Notch subunit heterodimerization and prevention of ligand-independent proteolytic activation depend, respectively, on a novel domain and the LNR repeats. Mol Cell Biol (2004) 24:9265–73. 10.1128/MCB.24.21.9265-9273.2004 15485896 PMC522238

[52] Brown MS Ye J Rawson RB Goldstein JL Regulated intramembrane proteolysis: a control mechanism conserved from bacteria to humans. Cell (2000) 100:391–8. 10.1016/S0092-8674(00)80675-3 10693756

[53] Struhl G Adachi A. Requirements for presenilin-dependent cleavage of Notch and other transmembrane proteins. Mol Cell (2000) 6:625–36. 10.1016/S1097-2765(00)00061-7 11030342

[54] Kopan R Ilagan MXG. The canonical Notch signaling pathway: unfolding the activation mechanism. Cell (2009) 137:216–33. 10.1016/j.cell.2009.03.045 19379690 PMC2827930

[55] Yamaguchi E Chiba S Kumano K Kunisato A Takahashi T Takahashi T Expression of Notch ligands, Jagged1, 2 and Delta1 in antigen presenting cells in mice. Immunol Lett (2002) 81:59–64. 10.1016/S0165-2478(01)00326-1 11841846

[56] Tanigaki K Han H Yamamoto N Tashiro K Ikegawa M Kuroda K Notch–RBP-J signaling is involved in cell fate determination of marginal zone B cells. Nat Immunol (2002) 3:443 10.1038/ni793 11967543

[57] Parks AL Klueg KM Stout JR Muskavitch MA. Ligand endocytosis drives receptor dissociation and activation in the Notch pathway. Development (2000) 127:1373–85. 10704384 10.1242/dev.127.7.1373

[58] Langridge PD Struhl G Epsin-dependent ligand endocytosis activates Notch by force. Cell (2018) 171:1383–96.e12. 10.1016/j.cell.2017.10.048 PMC6219616 29195077

[59] Nichols JT Miyamoto A Olsen SL D’Souza B Yao C Weinmaster G. DSL ligand endocytosis physically dissociates Notch1 heterodimers before activating proteolysis can occur. J Cell Biol (2007) 176:445–58. 10.1083/jcb.200609014 17296795 PMC2063980

[60] Chapman G Major JA Iyer K James AC Pursglove SE Moreau JLM Notch1 endocytosis is induced by ligand and is required for signal transduction. Biochim Biophys Acta (2016) 1863:166–77. 10.1016/j.bbamcr.2015.10.021 26522918

[61] Duojia P Rubin GM. Kuzbanian controls proteolytic processing of Notch and mediates lateral inhibition during *Drosophila* and vertebrate neurogenesis. Cell (1997) 90:271–80. 10.1016/S0092-8674(00)80335-9 9244301

[62] Sotillos S Roch F Campuzano S. The metalloprotease-disintegrin Kuzbanian participates in Notch activation during growth and patterning of *Drosophila* imaginal discs. Development (1997) 124:4769–79. 9428413 10.1242/dev.124.23.4769

[63] Lieber T Kidd S Young MW Lieber T Kidd S Young MW. Kuzbanian-mediated cleavage of *Drosophila* Notch. Genes Dev (2002) 16:209–21. 10.1101/gad.942302 11799064 PMC155326

[64] Brou C Logeat F Gupta N Bessia C LeBail O Doedens JR A novel proteolytic cleavage involved in Notch signaling: the role of the disintegrin-metalloprotease TACE. Mol Cell (2000) 5:207–16. 10.1016/S1097-2765(00)80417-7 10882063

[65] Mumm JS Schroeter EH Saxena MT Griesemer A Tian X Pan DJ A ligand-induced extracellular cleavage regulates gamma-secretase-like proteolytic activation of Notch1. Mol Cell (2000) 5:197–206. 10.1016/S1097-2765(00)80416-5 10882062

[66] Bozkulak EC Weinmaster G. Selective use of ADAM10 and ADAM17 in activation of Notch1 signaling. Mol Cell Biol (2009) 29:5679–95. 10.1128/MCB.00406-09 19704010 PMC2772745

[67] Thinakaran G Koo EH. Amyloid precursor protein trafficking, processing, and function. J Biol Chem (2008) 283:29615–9. 10.1074/jbc.R800019200 18650430 PMC2573065

[68] Shah S Lee S-F Tabuchi K Hao Y-H Yu C LaPlant Q Nicastrin functions as a gamma-secretase-substrate receptor. Cell (2005) 122:435–47. 10.1016/j.cell.2005.05.022 16096062

[69] Chyung JH Raper DM Selkoe DJ. Gamma-secretase exists on the plasma membrane as an intact complex that accepts substrates and effects intramembrane cleavage. J Biol Chem (2005) 280:4383–92. 10.1074/jbc.M409272200 15569674

[70] Hansson EM Stromberg K Bergstedt S Yu G Naslund J Lundkvist J Aph-1 interacts at the cell surface with proteins in the active gamma-secretase complex and membrane-tethered Notch. J Neurochem (2005) 92:1010–20. 10.1111/j.1471-4159.2004.02926.x 15715652

[71] Lah JJ Levey AI. Endogenous presenilin-1 targets to endocytic rather than biosynthetic compartments. Mol Cell Neurosci (2000) 16:111–26. 10.1006/mcne.2000.0861 10924255

[72] Urra S Escudero CA Ramos P Lisbona F Allende E Covarrubias P TrkA receptor activation by nerve growth factor induces shedding of the p75 neurotrophin receptor followed by endosomal gamma-secretase-mediated release of the p75 intracellular domain. J Biol Chem (2007) 282:7606–15. 10.1074/jbc.M610458200 17215246

[73] Zhang M Haapasalo A Kim DY Ingano LAM Pettingell WH Kovacs DM. Presenilin/gamma-secretase activity regulates protein clearance from the endocytic recycling compartment. FASEB J (2006) 20:1176–8. 10.1096/fj.05-5531fje 16645046

[74] Pasternak SH Bagshaw RD Guiral M Zhang S Ackerley CA Pak BJ Presenilin-1, nicastrin, amyloid precursor protein, and gamma-secretase activity are co-localized in the lysosomal membrane. J Biol Chem (2003) 278:26687–94. 10.1074/jbc.M304009200 12736250

[75] Vaccari T Lu H Kanwar R Fortini ME Bilder D. Endosomal entry regulates Notch receptor activation in *Drosophila melanogaster*. J Cell Biol (2008) 180:755–62. 10.1083/jcb.200708127 18299346 PMC2265571

[76] Gupta-Rossi N Six E LeBail O Logeat F Chastagner P Olry A Monoubiquitination and endocytosis direct γ-secretase cleavage of activated Notch receptor. J Cell Biol (2004) 166:73–83. 10.1083/jcb.200310098 15240571 PMC2172142

[77] Luty WH Rodeberg D Parness J Vyas YM. Antiparallel segregation of Notch components in the immunological synapse directs reciprocal signaling in allogeneic Th:DC conjugates. J Immunol (2007) 179:819–29. 10.4049/jimmunol.179.2.819 17617572

[78] Bache KG Slagsvold T Stenmark H. Defective downregulation of receptor tyrosine kinases in cancer. EMBO J (2004) 23:2707–12. 10.1038/sj.emboj.7600292 15229652 PMC514952

[79] Seto ES Bellen HJ Lloyd TE When cell biology meets development: endocytic regulation of signaling pathways. Genes Dev (2002) 16:1314–36. 10.1101/gad.989602 12050111

[80] Sorkin A von Zastrow M. Endocytosis and signalling: intertwining molecular networks. Nat Rev Mol Cell Biol (2009) 10:609–22. 10.1038/nrm2748 19696798 PMC2895425

[81] Doherty GJ McMahon HT. Mechanisms of endocytosis. Annu Rev Biochem (2009) 78:857–902. 10.1146/annurev.biochem.78.081307.110540 19317650

[82] Traub LM. Tickets to ride: selecting cargo for clathrin-regulated internalization. Nat Rev Mol Cell Biol (2009) 10:583–96. 10.1038/nrm2751 19696796

[83] Sen A Madhivanan K Mukherjee D Claudio Aguilar R. The epsin protein family: coordinators of endocytosis and signaling. Biomol Concepts (2012) 3:117–26. 10.1515/bmc-2011-0060 22942912 PMC3431554

[84] Praefcke GJK McMahon HT. The dynamin superfamily: universal membrane tubulation and fission molecules? Nat Rev Mol Cell Biol (2004) 5:133–47. 10.1038/nrm1313 15040446

[85] Trahey M Hay JC Transport vesicle uncoating: it’s later than you think. F1000 Biol Rep (2010) 6:1–6. 10.3410/B2-47 PMC2919759 20706600

[86] Sala E Ruggiero L Giacomo GD Cremona O Endocytosis in Notch Signaling Activation. Milano: Università Vita-Salute San Raffaele (2012). p. 12–4.

[87] Sorensen EB Conner SD gamma-secretase-dependent cleavage initiates Notch signaling from the plasma membrane. Traffic (2010) 11:1234–45. 10.1111/j.1600-0854.2010.01090.x 20573067 PMC2919600

[88] Chastagner P Israel A Brou C. AIP4/Itch regulates Notch receptor degradation in the absence of ligand. PLoS One (2008) 3:e2735. 10.1371/journal.pone.0002735 18628966 PMC2444042

[89] Sakata T Sakaguchi H Tsuda L Higashitani A Aigaki T Matsuno K *Drosophila* Nedd4 regulates endocytosis of Notch and suppresses its ligand-independent activation. Curr Biol (2004) 14:2228–36. 10.1016/j.cub.2004.12.028 15620649

[90] Berdnik D Torok T Gonzalez-Gaitan M Knoblich JA. The endocytic protein alpha-adaptin is required for numb-mediated asymmetric cell division in *Drosophila*. Dev Cell (2002) 3:221–31. 10.1016/S1534-5807(02)00215-0 12194853

[91] Seugnet L Simpson P Haenlin M. Requirement for dynamin during Notch signaling in *Drosophila* neurogenesis. Dev Biol (1997) 192:585–98. 10.1006/dbio.1997.8723 9441691

[92] Yan Y Denef N Schüpbach T. The vacuolar proton pump, V-ATPase, is required for Notch signaling and endosomal trafficking in *Drosophila*. Dev Cell (2009) 17:387–402. 10.1016/j.devcel.2009.07.001 19758563 PMC2758249

[93] Vaccari T Duchi S Cortese K Tacchetti C Bilder D. The vacuolar ATPase is required for physiological as well as pathological activation of the Notch receptor. Development (2010) 137:1825–32. 10.1242/dev.045484 20460366 PMC2867318

[94] Kobia F Duchi S Deflorian G Vaccari T. Pharmacologic inhibition of vacuolar H+ ATPase reduces physiologic and oncogenic Notch signaling. Mol Oncol (2014) 8:207–20. 10.1016/j.molonc.2013.11.002 24309677 PMC5528540

[95] Yamada K Fuwa TJ Ayukawa T Tanaka T Nakamura A Wilkin MB Roles of *Drosophila* Deltex in Notch receptor endocytic trafficking and activation. Genes Cells (2011) 16:261–72. 10.1111/j.1365-2443.2011.01488.x 21299753

[96] Hori K Fostier M Ito M Fuwa TJ Go MJ Okano H *Drosophila* deltex mediates suppressor of hairless-independent and late-endosomal activation of Notch signaling. Development (2004) 131:5527–37. 10.1242/dev.01448 15496440

[97] Diederich RJ Matsuno K Hing H Artavanis-Tsakonas S. Cytosolic interaction between deltex and Notch ankyrin repeats implicates deltex in the Notch signaling pathway. Development (1994) 120:473–81. 8162848 10.1242/dev.120.3.473

[98] Matsuno K Eastman D Mitsiades T Quinn AM Carcanciu ML Ordentlich P Human deltex is a conserved regulator of Notch signalling. Nat Genet (1998) 19:74–8. 10.1038/ng0598-74 9590294

[99] Boehm M Bonifacino JS. Genetic analyses of adaptin function from yeast to mammals. Gene (2002) 286:175–86. 10.1016/S0378-1119(02)00422-5 11943473

[100] Bröcker C Kuhlee A Gatsogiannis C Balderhaar HJ Hönscher C Engelbrecht-Vandré S Molecular architecture of the multisubunit homotypic fusion and vacuole protein sorting (HOPS) tethering complex. Proc Natl Acad Sci U S A (2012) 109:1991–6. 10.1073/pnas.1117797109 22308417 PMC3277535

[101] Wilkin M Tongngok P Gensch N Clemence S Motoki M Yamada K *Drosophila* HOPS and AP-3 complex genes are required for a deltex-regulated activation of Notch in the endosomal trafficking pathway. Dev Cell (2008) 15:762–72. 10.1016/j.devcel.2008.09.002 19000840

[102] Lehar SM Bevan MJ. T cells develop normally in the absence of both Deltex1 and Deltex2. Mol Cell Biol (2006) 26:7358–71. 10.1128/MCB.00149-06 16923970 PMC1636852

[103] Koch U Fiorini E Benedito R Besseyrias V Schuster-Gossler K Pierres M Delta-like 4 is the essential, nonredundant ligand for Notch1 during thymic T cell lineage commitment. J Exp Med (2008) 205:2515–23. 10.1084/jem.20080829 18824585 PMC2571927

[104] Palmer WH Deng WM. Ligand-independent mechanisms of Notch activity. Trends Cell Biol (2015) 25:697–707. 10.1016/j.tcb.2015.07.010 26437585 PMC4628868

[105] De Strooper B Annaert W Cupers P Saftig P Craessaerts K Mumm JS A presenilin-1-dependent gamma-secretase-like protease mediates release of Notch intracellular domain. Nature (1999) 398:518–22. 10.1038/19083 10206645

[106] Scott CC Gruenberg J. Ion flux and the function of endosomes and lysosomes: pH is just the start: the flux of ions across endosomal membranes influences endosome function not only through regulation of the luminal pH. Bioessays (2011) 33:103–10. 10.1002/bies.201000108 21140470

[107] Tian X Gala U Zhang Y Shang W Nagarkar Jaiswal S di Ronza A A voltage-gated calcium channel regulates lysosomal fusion with endosomes and autophagosomes and is required for neuronal homeostasis. PLoS Biol (2015) 13:e1002103. 10.1371/journal.pbio.1002103 25811491 PMC4374850

[108] Ebsen H Schröder A Kabelitz D Janssen O. Differential surface expression of ADAM10 and ADAM17 on human T lymphocytes and tumor cells. PLoS One (2013) 8:e76853. 10.1371/journal.pone.0076853 24130797 PMC3793918

[109] Skovronsky DM Moore DB Milla ME Doms RW Lee VM. Protein kinase C-dependent alpha-secretase competes with beta-secretase for cleavage of amyloid-beta precursor protein in the trans-Golgi network. J Biol Chem (2000) 275:2568–75. 10.1074/jbc.275.4.2568 10644715

[110] Chastagner P Rubinstein E Brou C. Ligand-activated Notch undergoes DTX4-mediated ubiquitylation and bilateral endocytosis before ADAM10 processing. Sci Signal (2017) 10:eaag2989. 10.1126/scisignal.aag2989 28611181

[111] Pearce LR Komander D Alessi DR. The nuts and bolts of AGC protein kinases. Nat Rev Mol Cell Biol (2010) 11:9–22. 10.1038/nrm2822 20027184

[112] Britton GJ Ambler R Clark DJ Hill EV Tunbridge HM McNally KE PKCθ links proximal T cell and Notch signaling through localized regulation of the actin cytoskeleton. Elife (2017) 6:e20003 10.7554/eLife.20003 28112644 PMC5310840

[113] Krummel MF Bartumeus F Gérard A. T cell migration, search strategies and mechanisms. Nat Rev Immunol (2016) 16:193–201. 10.1038/nri.2015.16 26852928 PMC4869523

